# Trends in scientific editing and good research practices: what do researchers-nurses know?

**DOI:** 10.1590/1980-220X-REEUSP-2021-0393

**Published:** 2022-01-05

**Authors:** Álvaro Francisco Lopes de Sousa, Maria Helena Palucci Marziale, Evelin Capellari Cárnio, Carla Aparecida Arena Ventura, Sara Soares Santos, Isabel Amélia Costa Mendes

**Affiliations:** 1 Centro Universitário UNINOVAFAPI, Teresina, PI, Brazil.; 2 Universidade Nova de Lisboa, Instituto de Higiene e Medicina Tropical, Global Health and Tropical Medicine, Lisboa, Portugal.; 3 Universidade de São Paulo, Escola de Enfermagem de Ribeirão Preto, Ribeirão Preto, SP, Brazil.

**Keywords:** Nursing, Research Personnel, Research, Publishing, Scholarly Communication, Knowledge, Enfermería, Investigadores, Investigación, Edición, Comunicación Académica, Conocimiento, Enfermagem, Pesquisadores, Pesquisa, Editoração, Comunicação Acadêmica, Conhecimento

## Abstract

**Objective:**

To verify researchers-nurses’ knowledge about trends in scientific publishing and good research practices.

**Method:**

A descriptive study carried out through an online survey with 197 nurses holding master’s and/or doctoral degrees from all Brazilian regions. To raise knowledge, a validated, self-administered and anonymous questionnaire with 18 questions on the subject was used. Descriptive and inferential analyzes were performed on researchers’ scores (Mann-Whitney test).

**Results:**

Among the specific questions, the mean of correct answers was 7.1: 6.4 for master’s and 7.4 for doctoral degree holders. There was a significant difference in the mean of correct answers between masters and doctors (p = 0.025), and between productivity scholarship holders and non-scholarship holders (p = 0.021), according to mean difference tests. Questions about predatory editorial practices were those in which researchers had the worst knowledge.

**Conclusion:**

We identified that, regardless of the education level (master’s or doctoral degree), nurses have little knowledge about the topics studied, which can compromise the quality of production and the scientific vehicles used to disseminate this knowledge.

## RESUMO

**Objetivo:**

Verificar o conhecimento de pesquisadores-enfermeiros sobre tendências em editoração científica e boas práticas em pesquisa.

**Método:**

Estudo descritivo, realizado por inquérito*online*com 197 enfermeiros com título de mestre e/ou doutor de todas as regiões brasileiras. Para levantar o conhecimento, utilizou-se um questionário validado, autoaplicável e anônimo com 18 questões sobre o assunto. Sobre o escore dos pesquisadores, realizaram-se análises descritivas e inferenciais (Teste de Mann-Whitney).

**Resultados:**

Entre as questões específicas, a média de acertos foi de 7,1, sendo 6,4 para mestres e 7,4 para doutores. Houve diferença significativa na média de acertos entre mestres e doutores (p = 0,025), e entre bolsistas e não bolsistas de produtividade (p = 0,021), segundo testes de diferença de médias. As questões sobre práticas editoriais predatórias foram aquelas em que os pesquisadores apresentaram pior conhecimento.

**Conclusão:**

Identificamos que, independente do grau de formação (mestrado ou doutorado), os enfermeiros têm baixo conhecimento sobre os temas estudados, o que pode comprometer a qualidade da produção e dos veículos científicos utilizados para disseminação desse conhecimento.

## RESUMEN

**Objetivo:**

Verificar el conocimiento de investigadores-enfermeros sobre las tendencias en la publicación científica y las buenas prácticas de investigación.

**Método:**

Estudio descriptivo, realizado a través de una encuesta en línea con 197 enfermeros con maestría y/o doctorado de todas las regiones brasileñas. Para aumentar el conocimiento se utilizó un cuestionario validado, autoadministrado y anónimo con 18 preguntas sobre el tema. Sobre la puntuación de los investigadores, se realizaron análisis descriptivos e inferenciales (prueba de Mann-Whitney).

**Resultados:**

Entre las preguntas específicas, la media de aciertos fue de 7,1, siendo 6,4 para los másteres y 7,4 para los médicos. Hubo diferencia significativa en la media de aciertos entre maestros y doctores (p = 0,025), y entre becarios de productividad y no académicos (p = 0,021), según pruebas de diferencia de medias. Las preguntas sobre prácticas editoriales depredadoras eran aquellas en las que los investigadores tenían el peor conocimiento.

**Conclusión:**

Identificamos que, independientemente del nivel de formación (maestría o doctorado), los enfermeros tienen poco conocimiento sobre los temas estudiados, lo que puede comprometer la calidad de la producción y los vehículos científicos utilizados para difundir este conocimiento.

## INTRODUCTION

Dissemination of research findings is one of the researcher’s duties, who can use various ways to disseminate them, such as publication in scientific journals, blogs, institutional website electronic pages, conventional and digital media (Facebook, Instagram, Twitter), e-books, books, etc.^(1–2)^. However, the publication of articles in peer-reviewed scientific journals remains the main means of dissemination recognized by academia, society as well as funding institutions. They are indicators used in national and international rankings of scientific production and technological innovation^(3)^.

Given the constant advancement of information technologies used in knowledge communication and dissemination, demands related to the management and editing of scientific journals that allow the advancement and modernization of the publication system are presented to journals that seek to adapt them to their editorial policies^(4–5)^. Likewise, such changes affect the modus operandi of authors and experts who issue opinions, and they may face challenges in mastering new concepts and complex tools with which they are not always familiar^(5,6,7)^.

This constant process of change still takes place in an environment of pressure on researchers to obtain high levels of productivity and hypercompetition for research funds, which can compromise scientific progress and increase unethical actions due to the relativization of integrity in the development and communication of scientific research^(8)^. The consolidation of predatory journals^(9)^, salami science^(10)^ and unethical actions that harm the integrity of the process such as plagiarism are among the internationally recognized practices that cause distrust in the credibility of scientists and in the editorial system, putting at risk the reliability of the scientific method and of science as a whole.

In this scenario of changes in the management of scientific publishing and pressure on authors to adapt to such changes, research institutions and funding agencies around the world have been encouraging the creation of guidelines for what has been called “good research practices”^(11–14)^: basic criteria that seek to promote and maintain ethical and quality standards for conducting, assessing and disseminating research that ensure the good exercise of scientific practice. However, studies are needed to demonstrate researchers’ knowledge in different areas about these “new processes and trends”, especially due to the lack of systematic assessment models that guide compliance with the precepts of good scientific practices, in addition to the considerable scarcity of studies in the world literature on this topic^(14)^.

Specifically with regard to nursing, investigations into knowledge on topics related to good practices in research and scientific editing are needed^(11,13–14)^. Nursing is a productive area worldwide; therefore, having a correct and up-to-date knowledge of concepts, trends and processes related to management in scientific publishing collaborates with the maintenance of scientific integrity, since it provides authors with a theoretical framework not only to avoid bad scientific practices, but also to promote, lead teams and apply good practices in publishing.

In this context, we seek to answer the following question: taking into account the intersections between new processes and trends in scientific publishing and the need to practice and promote good research practices, what is Brazilian researchers-nurses’ knowledge about these themes and their implications? Based on this, this study aimed to verify researchers-nurses’ knowledge about trends in scientific publishing and good research practices.

## METHOD

### Design of Study

This is a descriptive, analytical study, carried out through an online survey with nurses from all five administrative regions in Brazil.

### Population and Sample

The project adopted the acronym*Rn_in_Science*and its research universe is researchers-nurses with a master’s and/or doctoral degree, registered on the Lattes platform managed by the Brazilian National Council for Scientific and Technological Development (CNPq –*Conselho Nacional de Desenvolvimento Científico e Tecnológico*) of Brazil. To delimit the sample, the universe of master’s and doctoral degree holders in nursing provided by the Ministry of Education of Brazil was considered^(15)^, adopting a 95% confidence interval and a margin of error of 0.70 and using the formula for finite population, calculated in G.Power, requiring 180 participants.

For data collection, we used nurses registered in the Iberoamerican Forum for Scientific Publishing in Nursing (2020 edition), an international, free event held for over a decade that brings together researchers from all over Brazil. The forum had 1,056 participants, of which 737 who had a master’s and/or doctoral degree were contacted for participation. In addition, the group of 185 Research Productivity grant holders from CNPq, the Brazilian research funding agency, effective in 2020 were included., since these are the highly productive doctor nurses who stand out among their peers for their maturity and differentiated scientific production according to adopted normative criteria. The contact for the participation in the research of the two groups took place before the event took place to avoid selection or sampling biases.

### Inclusion and Exclusion Criteria

Researchers-nurses with master’s and/or doctoral degrees were included. Professionals who, although carrying out research in nursing, trained in other areas (psychology, biology, law, collective health and medicine), following the study objectives, in addition to professionals from other countries, were excluded.

### Data Collection

Data collection took place in April, May and June 2020 through a form hosted online on Google Forms, a free tool in the Google Docs application package, which allows to create a questionnaire and make forms available online^(16)^. This platform was chosen because of its gratuitousness, ease of use and research simplicity. For each participant, three contact attempts were made, resulting in 197 participants.

In this study, we used a questionnaire created by the authors themselves based on good research practice protocols^(12)^and editing guides from the International Committee of Medical Journal Editors (ICMJE). The questionnaire was face-content validated by five researchers holding doctoral degree with expertise in the subject, using the Delphi technique^(17)^, an efficient and consolidated methodology to generate consensus based on experts’ opinion on the subject. Nurses or librarians, with a doctoral degree and an h-index equal to or greater than 10 on the Web of Science indicating academic maturity/experience were chosen to compose the expert committee. The choice was made for convenience. The committee was composed of three nurses and two librarians, all with master’s and doctoral degrees.

The questionnaire was made available to the group of researchers online and assessed as to the degree of importance of each question for the research object using a Likert-type scale (1 – very small, 2 – small, 3 – reasonable, 4 – large and 5 – very large). Two rounds were carried out until the establishment of a consensus. The content validity coefficient (CVC) was used to analyze the agreement index so that, to remain on the form, the question needed to reach a minimum percentage of 0.8 of agreement^(18)^, percentage fulfilled by all items. Subsequently, the questionnaire was tested (pre-test) with 3 participants from the reference population, with no need to change.

The validated questionnaire was self-administered, anonymous, composed of seven sections: the first section (1) covered social, demographic and information related to researchers’ performance and training. The second section (2) was about productivity, peer review activities and editorials. The following sections address the specific contents of the object of this study with data related to: (3) scientometrics; (4) publishing templates; (5) journals and publishers; (6) good research practices; (7) plagiarism and self-plagiarism. The specific and multiple-choice questions totaled 18, each with four answer options, of which only one was correct.

### Data Analysis and Treatment

Data were analyzed using the Statistical Package for Social Sciences (SPSS) for Windows, version 27.0. Descriptive analyzes were performed, through the distribution of absolute frequencies, simple percentages and measures of central tendency and dispersion. For comparison purposes, professionals were divided into master’s and doctoral degree holders, and later between and productivity scholarship holders versus non-scholarship holders, in order to compare the mean of correct answers between groups. Research productivity scholarship holders are nurses holding a doctoral degree who stand out among their peers for their maturity and differentiated scientific production according to normative criteria.

After proving the non-normal distribution of the sample, through a test of adherence to normality applied to numerical variables (age and training time), the non-parametric Mann-Whitney test was used to test the hypotheses that the mean of correct answers was equal between groups. A p-value was adopted with a significance level of 0.05, so that, if p-value ≤ 0.05, the difference between the means was considered statistically significant.

### Ethical Aspects

This research was carried out in accordance with the recommendations contained in Resolution 466/12 of the Brazilian National Health Council (*Conselho Nacional de Saúde*), which brings together the ethical aspects of research involving human beings. The project was approved by the Research Ethics Committee of the*Universidade de São Paulo*at*Escola de Enfermagem de Ribeirão Preto*, under Protocol 3.833.855/2020. Research participants and judges signed an online Informed Consent Form. There was no conflict of interest between the responding researchers and collaborators.

## RESULTS

Among the 197 nurses participating, the mean age was 44 years (standard deviation (SD): 12.9; median: 43.0; min: 22; max: 74), the mean length of work was 19.8 years (SD: 13.1; median: 19.0; min: 1; max: 56), while training time had a mean of 20 years (SD: 13.1; median: 19.0; min: 1; max: 56). Other social, demographic, training and productivity characteristics are shown in Table 1.

**Table 1.** Sociodemographic, training and productivity characteristics of researchers-nurses (n: 197). Ribeirão Preto, SP, Brazil, 2020.

Variable	n	%
**Gender**		
Female	156	79.2
Male	41	20.8
**Age (years)**		
≤30 years	36	18.3
>30 years	161	81.7
Training time		
5 years or less	30	15.2
6–15 years	59	29.9
16 years and more	108	54.8
Maximum degree		
Master’s degree	50	25.4
Doctoral degree	147	74.6
Region of Brazil		
Midwest	8	4.1
Northeast	58	29.4
North	2	1.0
Southeast	96	48.7
South	33	16.8
Productivity scholarship		
Yes	68	34.5
No	129	65.5
Number of articles published*		
5 or less	42	21.3
6–15	47	23.9
16 and more	108	54.8
Funding grant*		
Yes	106	53.8
No	91	46.2
Editorial board member of scientific journals*		
Yes	85	43.1
No	112	56.9
Reviewer of scientific journals		
Yes	155	78.7
No	42	21.3

*In the last five years.

Table 2 shows the number of correct and wrong answers by researchers regarding the specific questions of the study. Overall, of the 18 questions, the mean of correct answers was 7.1 (SD: 2.6; median: 7.0), with the minimum recorded for one correct question (01), and the maximum, 15.

**Table 2.** Number of correct and wrong answers by researchers-nurses (n: 197) on specific questions related to good practices in research and scientific editing. Ribeirão Preto, SP, Brazil, 2020.

Specific questions	Master’s degree holders		Doctoral degree holders		Total	
	n	%	n	%	n	%
**1. Metrics – What is a researcher’s h-index?**						
Correct answers	29	58.0	98	66.7	127	64.5
Wrong answers	21	42.0	49	33.3	70	35.5
**2. What is CiteScore?**						
Correct answers	12	24.0	75	51.0	87	44.2
Wrong answers	38	76.0	72	49.0	110	55.8
**3. What is an impact factor?**						
Correct answers	25	50.0	81	55.1	106	53.8
Wrong answers	25	50.0	61	44.9	91	46.2
**4. What is i10-index?**						
Correct answers	20	40.0	62	42.2	82	41.6
Wrong answers	30	60.0	85	57.8	115	58.4
**5. What is rolling pass?**						
Correct answers	17	34.0	36	24.5	53	26.9
Wrong answers	33	66.0	111	75.5	144	73.1
**6. What is ahead of print publishing?**						
Correct answers	15	30.0	52	35.4	67	34.0
Wrong answers	35	70.0	95	64.5	130	66.0
**7. What are preprints?**						
Correct answers	23	46.0	72	49.0	95	48.2
Wrong answers	27	54.0	75	51.0	102	51.8
**8. What is salami science?**						
Correct answers	23	46.0	72	49.0	95	48.2
Wrong answers	27	54.0	75	51.0	102	51.8
**10. What can indicate that a journal/publisher is predatory?**						
Correct answers	32	64.0	97	66.0	129	65.5
Wrong answers	18	36.0	50	34.0	68	34.5
**11. Can predatory journals be indexed?**						
Correct answers	12	24.0	27	18.4	39	19.8
Wrong answers	38	76.0	120	81.6	158	80.2
**12. Can predatory journals have Qualis/CAPES?**						
Correct answers	21	42.0	40	27.2	61	31.0
Wrong answers	29	58.0	107	72.8	136	69.0
**13. Is there a list of predatory journals?**						
Correct answers	16	32.0	59	40.1	75	38.1
Wrong answers	34	68.0	88	59.9	122	61.9
**14. Among the examples below, data fabrication can be characterized as:**						
Correct answers	23	46.0	63	42.9	86	43.7
Wrong answers	27	54.0	84	57.1	111	56.3
**15. Among the examples below, the following may be considered a forgery:**						
Correct answers	15	30.0	40	27.2	55	27.9
Wrong answers	35	70.0	107	72.8	142	72.1
**16. The following justify authorship in a manuscript, according to the ICMJE recommendations:**						
Correct answers	19	38.0	73	49.7	92	46.7
Wrong answers	31	62.0	74	50.3	105	53.3
**17. The following can be considered plagiarism:**						
Correct answers	12	24.0	42	28.6	54	27.4
Wrong answers	38	76.0	105	71.4	143	72.6
**18. The following can be considered self-plagiarism:**						
Correct answers	30	60.0	73	49.7	103	52.3
Wrong answers	20	40.0	74	50.3	94	47.7

The question in which professionals performed the best was question 10 (which may indicate that a journal/publisher is predatory?) (65.5%) and the one with the lowest performance was question 11 (predatory journals may be indexed in bases?) (19.8%).

When we compared the mean of correct answers between master’s and doctoral degree holders (p = 0.025), and between productivity scholarship and non-productivity scholarship holders (p = 0.021), there was a statistically significant difference. Among those researchers who had only a master’s degree, the mean of correct answers was 6.4 (SD: 2.7; median: 6.0; min: 2; max: 13), while among those who had a doctoral degree, the mean of correct answers was slightly higher than 7.4 (SD: 2.5; median: 8.0; min: 1; max: 15).

## DISCUSSION

In this study, researcher-nurses had low knowledge on issues related to the recognition and use of information on good practices in research and scientific editing. We emphasize that not even the degree (doctoral) or the attribution of a research productivity scholarship (incentives based on productivity) were determining factors for a higher knowledge score on the concepts investigated in this study. From this perspective, the data are surprising, as they can directly imply the growth and improvement of Brazilian nursing as a science, as well as the quality of production and scientific vehicles used to disseminate this knowledge. The gap^(14)^existing in the literature related to studies that address the topic gives even greater importance to the data obtained, indicating a clear need for investment and targeting of actions aimed at familiarizing researchers-nurses with topics related to good practices in research and scientific editing.

It is important to emphasize that researchers are responsible for the advancement of science and do so through the conception, proposition and carrying out of research, communication of its results and cooperation and mentoring relationships with other scientists and researchers. Thus, researchers must lead with competence and useful knowledge that allows them to act in the best way^(12)^. Researchers’ ethical and responsible performance is, therefore, intrinsically related to their knowledge of good research practices, resulting in greater security in all stages of the process, from its conception to the translation phase of the knowledge produced^(13)^. In this way, the participants’ unsatisfactory performance, regardless of their degree (master’s or doctoral degree holders), demonstrates that qualified researchers, with an excellent mean of publications, long training time and even working on the editorial board of journals, may find it difficult to learn about products and processes in the current overview of scientific publishing.

Constant editorial demands are proposed to scientific journals and authors^(5)^. As an example of this, aspects of scientometrics investigated in this study show that researchers still have difficulty in recognizing the metrics of science, even those already consolidated. Scientometrics is related to the demography of the scientific community and has been used mainly to better distribute science support funds in developed and developing countries^(19)^. In this sense, indicators and metrics provide quantitative measures to measure activities, inputs and results of research, development and innovation, analyzing and comparing countries, universities, journals and researchers^(20)^. The gaps in researchers’ knowledge about this universe certainly imply limitations as to their possibilities of effectively participating in decisions, especially regarding changes in existing requirements and demands.

It is noteworthy, in this scenario, that most indicators and metrics questioned in the study have existed for a long time. The question referring to i10-index was the one that had the least amount of correct answers (41.6%) in the section referring to metrics. On the other hand, the greatest amount of correct answers in that same section was given to the question regarding h-index (64.5%). This finding is particularly interesting, as one index is derived from the other^(21)^. H-index, created in 2005, refers to the number of articles with citations greater than or equal to the same number, while i10-index, created by Scholar Google^(22)^, measures the number of publications with at least 10 citations in this database^(23)^.

With regard to publication models, mistakes were found in the question regarding continuous publication or rolling pass, with almost two thirds of wrong answers. This publication model refers to internet entry into desktop publishing dynamics, to facilitate editorial and publication processes, in addition to providing greater visibility to published articles. This method is characterized by the publication of articles in a single volume, without periodic breaks and waiting for the closing of one issue to publish another, which can increase the visibility of articles that gain greater possibility of consultations and citations^(24)^. On the other hand, the other older publication system, ahead of print, recorded about two-thirds of correct answers, which may indicate that, over time, authors may become more familiar with the topic and be able to recognize them.

Questions referring to salami science and preprints register correct answers from approximately half of our sample. The first term is old and goes back to the 1980s, referring to the practice of slicing up a single discovery, such as salami, to publish it with as many scientific articles as possible^(10,24–25)^. On the other hand, the term preprint has gained notoriety more recently with the advent of open science as a publishing model in which authors can make their texts available on servers before they are peer-reviewed^(25)^, with high adherence of nursing journals in Brazil and worldwide.

The questions about predatory editorial practices were those in which researchers showed the overall worst performance, especially in the question*Can predatory journals be indexed?*It is necessary for researchers to be able to recognize quality indexers and databases that have a systematic process of acceptance of a journal, not just based on fixed national criteria. The journal indexing process is almost always judicious and based on a series of indicators that validate good databases. Recognizing what these databases are and how they measure the quality and ethical aspects of the journals that compose them can prevent authors from being victims of predatory newspapers^(26)^.

Regarding the section on good research practices, the worst researchers’ performance was in the question that assessed the definition of forgery (27.9%). Among the various possibilities of scientific misconduct practices, data falsification deals with the manipulation of methods, equipment and processes that allow the alteration and/or omission of results, so as not to accurately represent the research^(27)^. This practice can still be divided into two: cooking, in which only the results that support the investigated hypothesis are kept and analyzed, and the data that could weaken it are omitted; and its more “discreet” form, trimming, which involves smoothing out data irregularity in order to make them more convincing for publication^(28)^.

Our data also show that researchers had greater difficulty in recognizing plagiarism (27.4%) than self-plagiarism (52.3%). This is an interesting finding, since, routinely, the literature places plagiarism at a higher level of severity than self-plagiarism. This is due to the consequence of the two practices, since the first involves a crime in relation to another author, and it can take different forms and intensities, from a literal copy to paraphrase, without the proper citation of the work that originated them^(29)^. Regardless of the type, the finding of bad scientific practices has harmful consequences to an area or to science as a whole, so that it is not enough to avoid scientific bad behavior, it is necessary to promote good behavior. For this, knowing and recognizing the different interfaces of these bad practices is necessary. No researcher shall facilitate, by action or omission, the occurrence or concealment of scientific misconduct^(16)^.

It is also worth highlighting the low percentage of correct answers, given the question about contributions that justify authorship in a scientific manuscript, with less than half (46.7%) of correct answers. For researchers, writing the project (42.6%) and data collection (8.6%) are sufficient criteria for authorship in a scientific manuscript. According to the ICMJE, those designated as authors of a manuscript must have substantially participated in its elaboration stages, recommending that authorship be based on the criteria: (a) substantial contributions to the work conception or design; (b) data collection, analysis and interpretation; (c) article writing or its critical review; (d) final approval of the version to be published^(30)^. Participants in this study had a high publication mean, and the finding that they have difficulties in listing sufficiently valid criteria for authorship may indicate that they are reproducing such practices in their manuscripts.

This research has limitations that must be considered. The option to use participants of an event in the area may have restricted the participation of other researchers-nurses, which hinders the ability for generalization. The platform used did not allow us to access the number of participants who accessed the form but chose not to respond, and it is not possible to measure losses. Although we have developed a data collection form validation process, the simplicity of the process can provide limited results, although validity and reliability have been ensured by the correct use of measures recognized in the literature^(18)^.

In the International Year of Nursing (2020), investigating and presenting indicators that support its consolidation as a science is extremely necessary, aiming at maintaining the production of quality knowledge in the area and having researchers in tune with the evolution of scientific communication practices, in order to apply in their own productions, form new teams of researchers, as well as contribute as journal reviewers or in other functions related to scientific editing. We believe that more investigations are needed, focused on understanding the occurrence of trends identified in this study in Brazilian nursing production.

## CONCLUSION

In our study, researcher-nurses, regardless of their level of education (master’s or doctoral degrees), had low knowledge on issues related to the recognition and use of information on good practices in research and scientific publishing. Our findings point to the need for strategies that identify weaknesses, strengthen gaps and expand knowledge, allowing the enrichment of researchers-nurses’ scientific training in topics related to good practices in research and scientific editing to qualify nursing production.
